# Analysis of Lipophilic Extractives from Fast Pyrolysis Bio-Oils

**DOI:** 10.1021/acs.energyfuels.1c04325

**Published:** 2022-05-16

**Authors:** Taina Ohra-aho, Maryam Ghalibaf, Raimo Alén, Christian Lindfors, Anja Oasmaa

**Affiliations:** † VTT Technical Research Centre of Finland Ltd, P.O. Box 1000, FI-02044 Espoo, Finland; ‡ Natural Resources Institute Finland (Luke), Tietotie 4, 31600 Jokioinen, Finland; § Laboratory of Applied Chemistry, Department of Chemistry, University of Jyväskylä, P.O. Box 35, FI-40014 Jyväskylä, Finland

## Abstract

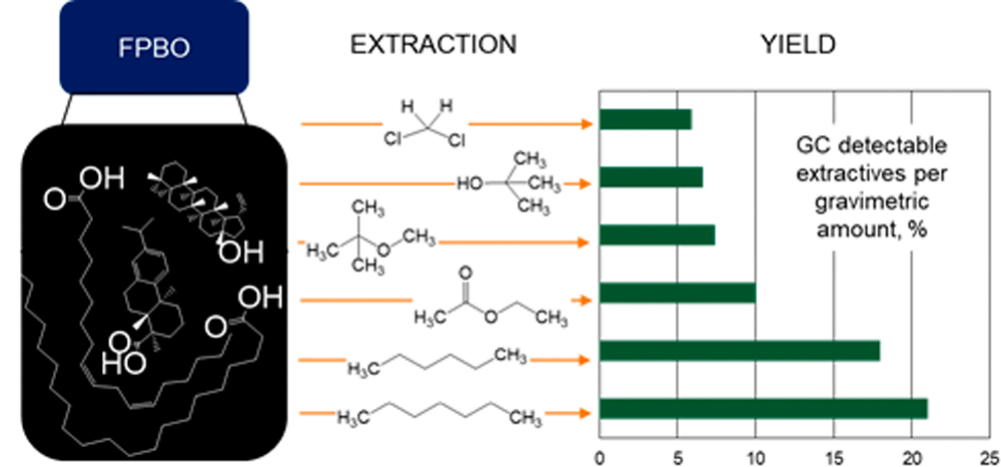

Fast pyrolysis bio-oils (FPBOs) originating from forest residues contain extractive-derived substances, which may form a separate, sticky layer with char particles on the surface of these bio-oils. In this study, first, the removal of extractive-derived substances from the bio-oil top phase was studied by common solvents with different polarities. In this case, the results indicated that when aimed at the maximum yield of single-phase fuel oil and thus the maximum amount of extractives removed, the use of *n*-heptane or *n*-hexane seems to be of benefit for this purpose. For safety reasons, the use of *n*-heptane was recommended. Second, an analysis practice (extraction time and the way of mixing) was optimized. In order to reduce the extraction time and enhance the extraction yield, it was important to break the oil surface in extraction. Third, based on the characterization results of the *n*-heptane extract by gas chromatography and ultraviolet spectroscopy, the detected compounds were classified as fatty acids, resin acids, esterified fatty acids, terpenoids, and steroids, and their total content (27 wt %) was lower than that of lignin-derived compounds (70 wt %). The extraction of the FPBO top phase with *n*-heptane followed by this analysis practice was a useful way to estimate the content and composition of lipophilic extractives.

## 1 Introduction

Wood-based fast pyrolysis is a near-mature technology (TRL 8–9) with production facilities in Finland [Fortum/Savon Voima in Joensuu, 30 MWth fast pyrolysis bio-oil (FPBO); Green Fuel Nordic in Lieksa, 15 MWth FPBO], in the Netherlands (Empyro/Twence in Hengelo, 15 MWth FPBO), and in Canada (Ensyn and Envergent, Renfrew 8 MWth FPBO and Côte Nord design capacity ∼30 MWth FPBO). One large-scale pyrolysis unit is under construction in Sweden (Pyrocell, Gävle) to produce FPBO to cofeed with vacuum gas oil in an existing oil refinery of Preem.^1−5^ Demonstration of FPBO for heating has been done,^6−8^ and standardization of FPBO as a boiler fuel has been carried out under the American Society for Testing and Materials and European Committee for Standardization guidelines.^9^ Quality specifications for the use of FPBO in industrial boilers (>1 MW h) are set by standard EN 16900-:2017.

In order to widen the feedstock base to less costly and sustainable waste materials, certain challenges have to be overcome. One of these obstacles is the inhomogeneity of FPBOs, especially in the case when feedstock, such as a forest residue with a high extractive content, is used. Due to the presence of needles and bark, a forest residue contains a significantly higher amount of extractives than stem wood.^10−12^ The composition and content of wood extractives are highly affected by the type, seasoning, and part of the wood (stem wood, bark, or needles).^11^ Wood extractives cover a large number of different compounds, which can be simply divided into hydrophilic and lipophilic (hydrophobic) compounds. Hydrophilic extractives are water-soluble and consist of carbohydrates and proteins. Lipophilic extractives can be extracted by means of a nonpolar solvent and can be divided into resins, fats, waxes, fatty acids, alcohols, steroids, and higher hydrocarbons.^13^ Lipophilic extractives are thermally more stable than hydrophilic extractives. However, some decarboxylation, dehydration, and ester bond scission may take place in pyrolysis during the vaporization.^14^ After condensation of pyrolysis vapors, particularly lipophilic extractive-derived substances tend to separate out from the highly polar bio-oil, forming a sticky layer on the surface of the FPBO.^11,15−18^ In addition, these sticky extractive-derived substances grasp the char particles when rising up to the surface.^11^ The extractive-rich top layer can be removed and used separately after the separation of char. The removal of char is of benefit in order to decrease the particulate emissions during burning if the bio-oil is used as a fuel.^17^

Based on earlier studies, lipophilic extractive-derived substances in FPBO are enriched with fatty acids, fatty alcohols, triglycerides, terpenes, and resin acids.^11^ The content and composition vary depending on the feedstock used for the production of FPBO. In order to efficiently remove and further utilize the extractive-rich fraction of FPBO, for example, as a fuel or chemicals, more information on its composition and amount is needed. The content of extractive-derived substances in FPBO has been determined as a weight percentage after *n*-hexane^11,17^ or toluene^19^ extraction, followed by solvent evaporation and quantification of the residue. However, a more detailed method description and justification for the choice of the solvent has not been reported. Thus, there is a need for a more precise study on the systematic evaluation of extractive-derived substances’ solubilities in various solvents. Detailed composition analysis of solvent extracts has been performed by gas chromatography (GC) combined with a mass selective detector (MSD) after silylation.^11,17,19^ Based on the results in the study by Oasmaa et al.,^11^ the GC-detectable compounds and the total yield of the *n*-hexane extract significantly varied, indicating that hexane also dissolves compounds not classified as extractive-derived compounds. In this study, the solubility of extractive-derived compounds in FPBO in various solvents was investigated, and extract composition was analyzed in detail. Thereafter, optimization of the extraction procedure was performed.

## 2 Experimental Section

### 2.1 Materials

FPBO was produced from forest residues consisting mainly of birch and aspen and a minor amount of softwood at around 500 °C with a residence time of less than 2 s and in the absence of oxygen using a VTT’s 20 kg/h pilot-scale pyrolyzer. After production, two phases were formed in the FPBO. The quantity of the top phase was 11 wt % of the oil product, and it was separated from the main product phase (bottom phase, 89 wt %) by scraping. Samples were divided into several containers and stored in a freezer. Analysis results of both the top and bottom phase compositions have been reported in detail elsewhere.^18^ Shortly, the chemical compositions of the top and bottom phases were (as wt %) water (19.5 and 24.4); sum of acids, aldehydes, ketones, alcohols, pyrans, and furans (19.4 and 23.7); sugar derivatives (21.9 and 28.8); lignin derivatives (23.1 and 16.4); extractives (16.4 and 2.8); and solids (2.9 and 0.04), respectively. The extractive-rich top phase of the FPBO was used for method development. The forest residue used for FPBO production was a mixture of several wood species; therefore, various lipophilic extractives were present in the bio-oil, more than the FPBO produced in a single biomass.

Solvents such as *n*-heptane (>99%, Merck), *n*-hexane (99%, VWR), methyl *tert*-butyl ether (MTBE, >99.8%, Sigma-Aldrich), ethyl acetate (EtOAc, 99.5%, Sigma-Aldrich), *tert*-butanol (*t*-BuOH, 99.5%, Merck), and dichloromethane (DCM, 99.8%, Aldrich) with a relative polarity of 0.012, 0.009, 0.124, 0.228, 0.389, and 0.309,^20,21^ respectively, were used. Betulinol (>97.5%, from Merck) was used as an internal standard.

### 2.2 Methods

#### 2.2.1 Extraction with Solvents

The extractive content of the top phase was determined by extracting a 1 ± 0.001 g sample with 30 mL of various solvents (*n*-heptane, *n*-hexane, MTBE, EtOAc, *t*-BuOH, and DCM) for 20 h using a shaker. The extract obtained was then dried before weighing by means of a gentle nitrogen gas stream.

#### 2.2.2 Optimization of Extraction

For the optimization of conditions, extraction of the top phase (1 g in an Erlenmeyer flask with a cap) was performed at room temperature as follows: in a shaker, at 140 rpm (INFORS TR-225), with and without a stir bar for 2, 4, 8, and 20 h with 30 mL of *n*-heptane; in a shaker with a stir bar for 24 h with 100 mL of *n*-heptane (for 20 h with 33 mL of *n*-heptane and then two times for 2 h with 33 mL of *n*-heptane); and for 2 h in an ultrasonic bath (SONO SWISS Typ SW 3 Nr 003764). In the latter case, the same original sample was extracted five times for 25 min using 20 mL of *n*-heptane. This was because the extraction temperature should not rise above 40 °C*.* After the extraction, the extract was removed by decanting into a tared glass vial. Thereafter, *n*-heptane was evaporated under nitrogen, and the yield was weighed. The number of extractions performed was four or five. We calculated the gravimetric solvent extraction yield of the FPBO sample according to eq 1
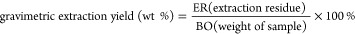
1where gravimetric extraction yield = amount of solvent-extractable compounds in the sample (the bio-oil top phase), wt %; ER = extraction residue after solvent extraction and evaporation of the solvent, grams; and BO = the amount of sample taken for the analysis, grams.

#### 2.2.3 Analysis

Extracted materials obtained with selected solvents were derivatized with a silylation reagent *N*,*O*-bis(trimethylsilyl)trifluoroacetamide (BSTF) with 1% of trimethylchlorosilane (TMCS). For the quantification, a known amount of the internal standard, betulinol, was added to the sample before silylation. The composition of extractives was determined using an Agilent 6850 Series gas chromatograph equipped with a Supelco Equity-5 column (30 m × 0.32 mm I.D. with 0.25 μm film thickness) and a flame ionization detector (FID). The FID was operated at 300 °C with a hydrogen flow and airflow of 40.0 and 450.0 mL/min, respectively. The column temperature program was 100 °C (1.5 min), 6 °C/min to 180 °C (10 min), and 4 °C/min to 290 °C (20 min). The amount of GC/FID-detectable compounds was quantified using betulinol as an internal standard. For the extractive identification, the same samples after derivatization were analyzed with an Agilent 6890 Series gas chromatograph equipped with a ZB-5HT Inferno column (30 m × 0.25 mm I.D. with 0.25 μm film thickness), by a gas chromatograph equipped with an MSD, and by applying the same chromatographic conditions as those used in the GC/FID. We calculated the GC-detectable compound yield after solvent extraction of the bio-oil top phase according to eq 2
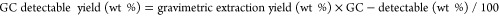
2where GC detectable yield = amount of GC-detectable compounds in the sample (bio-oil top phase), wt %. Gravimetric extraction yield = amount of solvent-extractable compounds in the sample (bio-oil top phase), wt %. GC – detectable = compounds detected by GC after solvent extraction, wt %.

**Figure 1:**
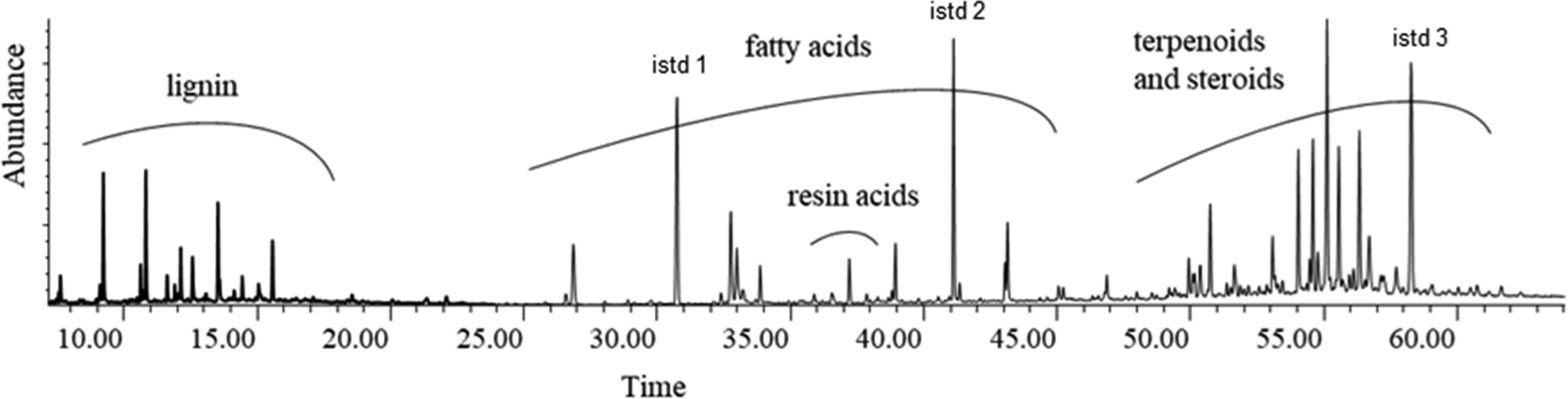
Chromatogram of the *n*-heptane extract of the top phase of FPBO after extraction for 20 h in a shaker. Internal standards marked in the chromatogram are heptadecanoic acid C_17:0_ (istd 1), heneicosanoic acid C_21:0_ (istd 2), and betulinol (istd 3).

As previously stated, for the composition analysis extracts from the optimization study were derivatized with a silylation reagent (BSTF +1% TMCS). These analyses were carried out using an Agilent 6890 series gas chromatograph equipped with an Agilent 5973 MSD and a capillary column of DB-5 (30 m × 0.25 mm I.D. with 0.33 μm film thickness). The column temperature program was 100 °C (1.5 min), 6 °C/min to 180 °C (10 min), and 4 °C/min to 300 °C (23 min) with a column flow rate of 1.2 mL/min (He). A mass spectrometry scan range of 30–800 (70 eV) was used. All samples were analyzed with and without the following internal standards: heptadecanoic acid C_17:0_ (istd 1), heneicosanoic acid C_21:0_ (istd 2), and betulinol (istd 3). Internal standards were added to the samples before silylation. The same samples were analyzed using an Agilent 7890 gas chromatograph/FID equipped with a capillary column of HP-5 (30 m × 0.32 mm I.D. with 0.25 μm film thickness). The column temperature program was 1.5 min at 100 °C, 6 °C/min to 180 °C (10 min), and 4 °C/min to 290 °C (25 min) using a column flow rate of 1.5 mL/min (H_2_). The FID was operated at 300 °C with a hydrogen flow and an airflow of 30 and 400 mL/min, respectively. In this case, lignin-derived compounds, fatty acids, resin acids, and terpenes together with terpenoids were integrated as a sum of peaks between selected retention times, as shown in Figure 1. Fatty acid internal standards (istd 1 and istd 2) were used to calculate the content of fatty acids, whereas betulinol was used to calculate the content of resin acids, lignin, terpenoid, and steroids. Equation 2 was used to calculate extractive-derived compound yield (wt %) in the sample.

Thermochemolysis to determine esterified fatty acids from the *n*-heptane extract (4 h extraction in a shaker with a stir bar) using two different reagents, tetramethyl ammonium hydroxide (TMAH) and tetramethyl ammonium acetate (TMAAc), was performed online in a Pyrolab pyrolyzer (Pyrolab 2000, Sweden) connected to an Agilent 7890B series gas chromatograph with a FID and an MSD (Agilent 5977A). A more detailed description of the method is shown elsewhere.^22^ About 0.1 mg of the sample with heneicosanoic acid as an internal standard and a derivatization reagent (TMAH or TMAAc) was mixed on the filament and thereafter pyrolyzed at 600 °C for 2 s. The products were separated in a gas chromatograph equipped with a midpolar capillary column DB-1701 (30 m × 0.25 mm I.D. with 1 μm film thickness). The column temperature program was 2 min at 80 °C, 8 °C/min to 160 °C, and 6 °C/min to 280 °C (5 min) with a column flow rate of 1 mL/min. The simultaneous detection of degradation products was done using a FID and an MSD. The FID was operated at 300 °C using a hydrogen flow and an airflow of 45 and 450 mL/min, respectively. Mass spectra were obtained using an electron ionizer (70 eV) and having a full scan mode in the mass range of 46–650 *m*/*z*. After the analysis of fatty acids, the content measured with both TMAH and TMAAc thermochemolysis methods was calculated using an internal standard. The yield of esterified fatty acids after solvent extraction of the bio-oil top phase was calculated as follows
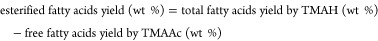
3

The content of lignin-derived substances in the *n*-heptane extraction residue (4-h extraction in a shaker with a stir bar) was determined using an ultraviolet (UV) spectrophotometer Hitachi U-2000 (Tokyo, Japan) at 280 nm after dissolving the extraction residue in an alkali solution (NaOH solution of 0.1 M) at room temperature. Before the analysis, the absorptivity value was determined for pyrolysis lignin (lignin-pyrolysis derivatives separated from FPBO by water extraction).^23^ Accurately 26.6 mg of pyrolysis lignin was dissolved in 1000 mL of alkali solution (0.1 M NaOH). The absorbance of 0.522 measured at 280 defines the absorptivity value of 20 L/(g cm) for pyrolysis lignin that was used to determine lignin-derived substance contents in the extraction residue. The absorbance was measured, and the lignin-derived substance content in the extraction residue was calculated using eq 4.
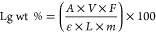
4where Lg wt % = lignin derivatives in the sample, wt %; *A* = absorbance of the sample; *V* = sample volume, *L*; *F* = dilution factor; ε = absorptivity value for pyrolysis lignin, 20 L/g cm; *L* = the length of the light path in the cuvette, 1 cm; and *m* = sample amount, grams.

#### 2.2.4 Statistical Analysis

The one-way analysis of variance (ANOVA; data analysis in Excel, Microsoft Office Professional Plus 2016) was used to determine whether there are any statistically significant differences between the means of five replicates of extractions performed for different times (2, 4, 8, and 20 h) in a shaker with a stir bar. The one-way ANOVA with a significance level of 0.05 was used to assess statistical significance.

## 3 Results and Discussion

### 3.1 FPBO Phases

**Figure 2:**
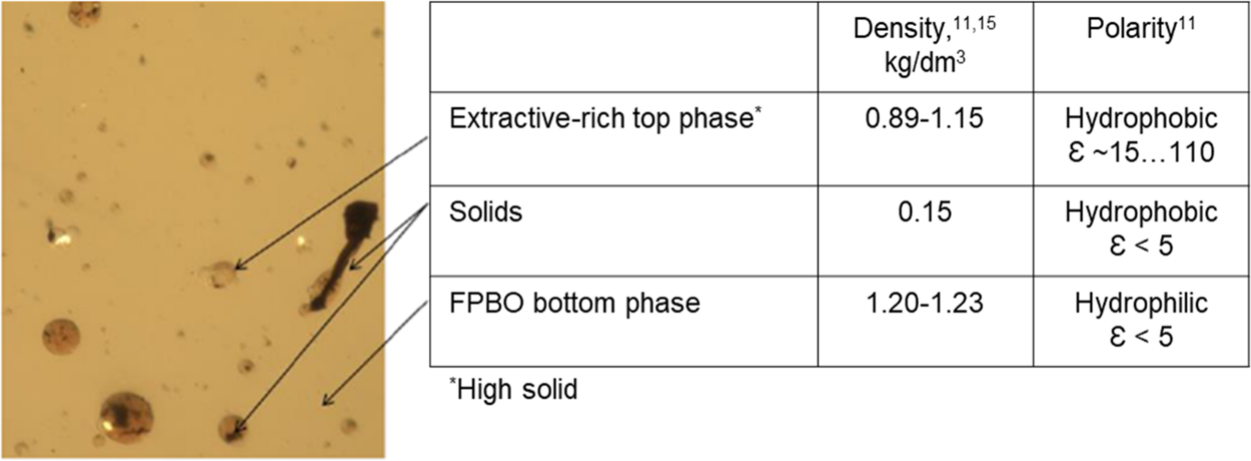
FPBO phases from optical microscopy analysis and literature values for the density and polarity of the fractions.^11,17^

It could be detected by optical microscopy that the forest residue-derived FPBO forms two phases with different polarities (Figure 2). The round-shape oily-like material (seen in Figure 2) forms the top layer, which is mainly composed of extractives enriched with solid materials. The main phase (the bottom phase) is mainly composed of hydrophilic lignin- and carbohydrate-derived compounds including acids, alcohols, aldehydes, ketones, furans, and pyrans.^18^ Since the extractive-rich top phase also contained the same compounds originating from lignin and carbohydrates, a selective solvent extraction was needed to separate extractive-derived substances from the other bio-oil compounds. In practice, there is a general need for a solvent with a low boiling point (<100 °C) that can efficiently remove lipophilic extractives.

### 3.2 Selection of the Solvent

Six solvents—*n*-hexane, *n*-heptane, MTBE, EtOAc, DCM, and *t*-BuOH—with different polarities were selected to remove extractives from the bio-oil top phase. Of these solvents, only *n*-hexane was used to determine lipophilic extractives from FPBO,^11,17^ whereas *n*-hexane and DCM were used to extract wood and bark^24−28^ and MTBE, DCM, and EtOAc for pulping effluents.^29^ Alcohols were mostly used for solid biomasses together with nonpolar solvents in a sequential extraction.^26^

Extraction yields of 15 to 81 wt % of the top phase (1 g) when using different solvents indicated that each solvent clearly has a different effect on the removal of materials from the top phase. Both the nonpolar solvents, *n*-heptane and *n*-hexane, gave similar yields, but the extraction yield was increased when more polar solvents were used: *n*-heptane ≈ *n*-hexane < MTBE < *t*-BuOH < DCM < EtOAc. However, the yield did not directly follow the relative polarity of the solvent.^20,21^ The highest extraction yield was obtained with EtOAc. It seemed that all other FPBO components than water (water content 19.5 wt %) were dissolved in EtOAc (Table 1). For the evaluation of the extractable materials, GC analyses were performed. As shown in Table 1, the solvent extracted materials were not completely detectable by GC. With respect to this, the total yield of GC-detectable compounds was the lowest for DCM (GC-detectable yield per gravimetric yield). Solvents with higher polarity (MTBE, EtOAc, *t*-BuOH, and DCM) gave higher gravimetric results; however, those fractions contained less GC-detectable compounds compared to those in *n*-heptane and *n*-hexane. This meant that the main portion of the extractable material was not GC-detectable.

**Table 1:** Gravimetric Extraction and GC-Detectable Compound Yields (wt % of the Top Phase) with Different Solvents

parameter	*n*-hexane	*n*-heptane	MTBE	EtOAc	DCM	*t*-BuOH
gravimetric yield	15 ± 1	15 ± 1	49 ± 2	81 ± 2	69 ± 2	60 ± 2
GC-detectable compounds	2.7 ± 0.2	3.1 ± 0.2	5.1 ± 0.2	6.0 ± 0.2	4.1 ± 0.2	4.0 ± 0.2
GC-detectable yield/gravimetric yield, %	18 ± 0	21 ± 2	10 ± 1	7.4 ± 0.4	5.9 ± 0.2	6.6 ± 0.1

Nonpolar solvents, *n*-hexane and *n*-heptane, dissolved extractives more selectively (fatty acids, resin acids, terpenoids, and sterols) than the other solvents (Table 2). More lignin monomers and anhydrosugars were detected in the MTBE, EtOAc, *t*-BuOH, and DCM extracts than in those obtained with *n*-heptane and *n*-hexane. These nonpolar solvents dissolved a slightly lower amount of extractives than MTBE, EtOAc, and DCM but a somewhat higher amount than *t*-BuOH. In cases of *n*-hexane and *n*-heptane, the composition of extractives was rather similar but varied among the other solvents. For example, resin acids were present in *n*-heptane, *n*-hexane, and DCM, but other solvents seemed not to be able to dissolve them. EtOAc had the best dissolving power for all other components except resin acids. Hence, it was not suitable for the bio-oils because it dissolved 80 wt % of the bio-oil top phase. This finding was the same for MTBE, *t*-BuOH, and DCM. In contrast, both *n*-heptane and *n*-hexane were more selective than other tested solvents for extractives. Slightly more extractives were dissolved in *n*-heptane than in *n*-hexane. For this reason, based on these results and the less toxic nature of *n*-heptane, *n*-heptane was recommended for this purpose instead of *n*-hexane.

**Table 2:** Compounds in the Top Phase Determined by GC after Solvent Extraction (wt %)

compound groups	*n*-hexane	*n*-heptane	MTBE	EtOAc	DCM	*t*-BuOH
fatty acids	0.87	0.95	1.18	0.99	1.10	0.69
tetradecanoic acid	0.02	0.02				
pentadecanoic acid	0.03	0.03				
hexadecanoic acid	0.13	0.10	0.17	0.19	0.19	0.13
heptadecanoic acid	0.02	0.02				
9,12-octadecanoic acid	0.17	0.17	0.19	0.35	0.37	0.19
oleic acid	0.15	0.15	0.18			
stearic acid	0.07	0.07	0.10	0.08	0.11	0.07
eicosanoic acid	0.05	0.09	0.11	0.07	0.06	0.06
heneicosanoic acid	0.05	0.06	0.06			
docosanol	0.02	0.03				
tetracosanoic acid	0.07	0.10	0.15	0.09	0.16	0.08
docosanoic acid	0.11	0.14	0.23	0.21	0.21	0.16
fatty acid esters	0.03	0.03	0.07	0.08		0.06
hexadecanoic acid butyl ester	0.03	0.03				
octadecanoic acid butyl ester			0.07	0.08		0.06
resin acids	0.07	0.06			0.06	
dehydroabietic acid	0.07	0.06			0.06	
terpenoids and steroids	0.77	0.75	0.94	1.03	0.78	0.71
stigmastan-3,5-diene	0.15	0.11	0.22	0.27		0.20
β-sitosterol	0.20	0.21	0.23	0.24	0.25	0.16
lupenone	0.42	0.43	0.49	0.52	0.53	0.35
lignin monomers	0.31	0.39	0.91	1.38	0.79	0.74
vanillin	0.02	0.04	0.06	0.20		0.14
4-propenylguaiacol	0.05	0.07	0.05	0.08		0.06
acetosyringone	0.05	0.05	0.11	0.14		0.12
syringol			0.06	0.06	0.07	0.05
4-methylsyringol	0.07	0.09	0.21	0.23	0.22	0.13
4-propenylsyringol	0.06	0.08	0.25	0.45	0.35	0.10
Syringaldehyde	0.06	0.07	0.17	0.21	0.16	0.14
Acetosyringone	0.05	0.05	0.11	0.14		0.12
Anhydrosugars			0.76	1.56	0.35	1.13
Arabinofuranose			0.08			
Levoglucosan			0.69	1.47	0.35	0.97
Galactopyranose				0.10		0.16
GC-detectable unidentified compounds	0.96	0.80	1.27	1.00	0.66	1.04

### 3.3 Extraction Method

For the optimization of the extraction time in extractions with *n*-heptane for 2, 4, 8, and 20 h, a shaker with and without a stir bar was used. In addition, the extraction for 24 h in a shaker with the stir bar using 100 mL of the solvent was compared with that in an ultrasonic bath for 2 h with the same amount of solvent.

**Figure 3:**
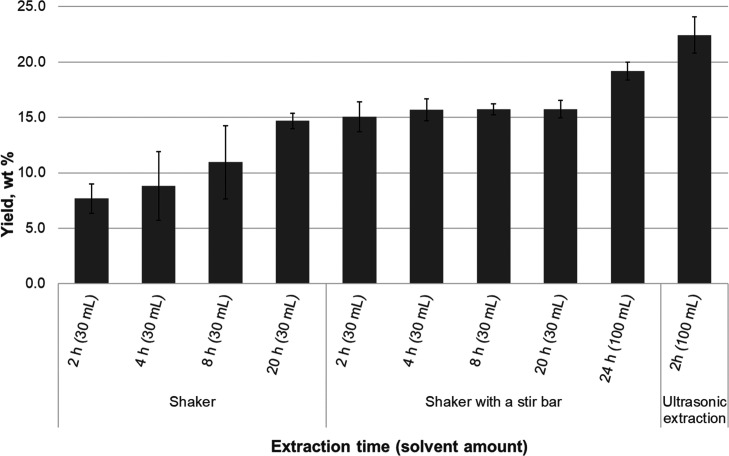
*n*-Heptane extraction yield under different conditions.

The extraction yield in a shaker without a stir bar increased with time (2–20 h), whereas with the stir bar, the yield was the same for all reaction times (Figure 3). The latter result was confirmed statistically using the one-way ANOVA test. Table 3 shows the output values of the ANOVA analysis and whether there is a statistically significant difference between the means of extraction yields obtained at different extraction times (*p* < 0.05). The significance value was 0.668, which was higher than 0.05. Therefore, there was no statistically significant difference between extraction yields at different extraction times (2, 4, 8, and 20 h). The stir bar broke the bio-oil surface that enhanced the solvent interaction with bio-oil components. This way the time needed for extraction was reduced, and the reproducibility was improved. In a shaker without a stir bar, the overnight extraction was needed to obtain good yields together with reproducible results. A small increase in yield was observed when the sample was washed twice with 30 mL of solvent after 20 h extraction. The greatest yield was obtained by using the ultrasonic extraction. However, heat was generated, which might not only enhance the solubility of bio-oil compounds in *n*-heptane but also facilitate the removal of compounds other than extractives (Table 4). The clear disadvantage was that the change of solvent was needed at intervals of 20 min to avoid excess heating. It was also observed that the change of solvent five times was needed to improve the reproducibility. The effect of the extraction time on the GC-detectable compounds was evaluated for the selected samples.

**Table 3:** One-Way ANOVA Results to Show the Effect of the Extraction Time (2, 4, 8, and 20 h) on the Extraction Yields (*n*-Heptane Extraction in a Shaker with a Stir Bar)^a^

source of variation	SS	df	MS	*F*	*P*-value	*F* crit
between groups	1.484	3	0.495	0.530	0.668	3.239
within groups	14.927	16	0.933			
total	16.411	19				

a The main difference is significant at the 0.05 level.

**Table 4:** Composition of *n*-Heptane Extracts in the Top Phase Determined by GC (wt %)

	Shaker with stir bar					ultrasonic extraction
compound groups	2 h (30 mL)	4 h (30 mL)	8 h (30 mL)	20 h (30 mL)	24 h (100 mL)	2 h (100 mL)
GC-detectable yield/gravimetric yield, %	25 ± 1	27 ± 3	26 ± 0	28 ± 1	26 ± 4	24 ± 1
extractives total	3.92 ± 0.18	4.25 ± 0.43	4.05 ± 0.03	4.41 ± 0.20	4.93 ± 0.78	5.23 ± 0.12
fatty acids	1.01 ± 0.04	1.07 ± 0.09	1.02 ± 0.01	1.16 ± 0.09	1.07 ± 0.08	1.56 ± 0.14
resin acids	0.05 ± 0.00	0.05 ± 0.00	0.05 ± 0.00	0.05 ± 0.00	0.06 ± 0.00	0.04 ± 0.00
terpenoids and steroids	2.86 ± 0.14	3.13 ± 0.34	2.98 ± 0.01	3.20 ± 0.11	3.78 ± 0.85	3.62 ± 0.26
lignin monomers	0.96 ± 0.06	0.91 ± 0.12	0.73 ± 0.11	0.73 ± 0.02	0.71 ± 0.27	0.56 ± 0.08

Based on the detailed GC analyses, the compositions of extracts were similar under different extraction conditions; only minor differences were observed in the yields of different compounds or compound groups (Table 4). A longer extraction time did not increase the yield of GC-detectable extractive per gravimetric yield. Both sequential extractions with a higher solvent volume enhanced the total yield of GC-detectable extractives, mainly terpenoids and steroids. However, GC-detectable extractive yield per gravimetric yield was at a similar level to one-batch extractions, which was the lowest in ultrasonic extraction. Several extraction steps dissolved more other components than the extractives. Thus, the simple extraction for 2 h in a shaker with a stir bar was sufficient to remove extractives from the bio-oil top phase.

**Table 5:** Fatty Acids from the *n*-Heptane Extraction Residue (4 h Extraction in a Shaker with a Stir Bar) by TMAH Thermochemolysis and TMAAc Thermochemolysis (wt %)

	free and esterified acids TMAH	free acids by TMAAc
fatty acids, total	1.22 ± 0.18	0.85 ± 0.17
branched fatty acids	0.09 ± 0.01	0.06 ± 0.01
octanoic acid	0.02 ± 0.00	0.00
nonanoic acid	0.01 ± 0.00	0.00
dodecanoic acid	0.01 ± 0.00	0.01 ± 0.00
tridecanoic acid	0.01 ± 0.00	0.00
tetradecanoic acid	0.03 ± 0.00	0.01 ± 0.00
pentadecanoic acid	0.03 ± 0.00	0.02 ± 0.00
hexadecanoic acid	0.20 ± 0.03	0.11 ± 0.02
oleic acid	0.13 ± 0.02	0.08 ± 0.02
linoleic acid	0.32 ± 0.05	0.21 ± 0.04
octadecanoic acid	0.08 ± 0.01	0.08 ± 0.02
nonadecanoic acid	0.02 ± 0.00	0.02 ± 0.02
eicosanoic acid	0.10 ± 0.02	0.09 ± 0.02
docosanoic acid	0.11 ± 0.02	0.09 ± 0.02
tricosanoic acid	0.03 ± 0.00	0.02 ± 0.00
tetracosanoic acid	0.05 ± 0.01	0.04 ± 0.01

### 3.4 Non-GC-Detectable Compounds

#### 3.4.1 Fatty Acid Esters

The difference between the total extractable material and the GC-detectable compounds was high. This practically meant that the *n*-heptane extracts contained high-molar-mass compounds, which were not detectable by GC. A minor amount of fatty acid esters was observed from the top phase of bio-oil (Table 2). Due to the size, many of the fatty acid esters, for example, triglycerides and steryl esters, were not detected by GC. Hence, thermochemolysis was used to determine these esterified fatty acids in the extraction residue (4 h extraction in a shaker with a stir bar). In this method, the esterified fatty acids can be distinguished from the free fatty acids using two reagents, TMAH and TMAAc.^30^ In thermochemolysis, organic substances are degraded into smaller fragments by means of a chemical reagent and heat. As a strong base, TMAH cleaves ether and ester bonds via hydrolytic scission at an elevated temperature. Finally, these reactions lead to the methylation of etherified and esterified functional groups, together with free alcohols, acids, and salts. As a neutral reagent, TMAAc is only capable of reacting with free acids, hydroxyl groups, and salts. A combination of these two reagents may distinguish bound fatty acids from free acids.

The results of fatty acids from the *n*-heptane extraction residue by TMAH thermochemolysis and TMAAc thermochemolysis are presented in Table 5. Based on the thermochemolysis analysis, the amount of the esterified fatty acids in the top phase of bio-oil was 0.38 ± 0.08 wt %. This was a somewhat higher value compared to that of the esterified fatty acids detected in the direct GC analysis. However, it could not explain a high difference between the total extraction yield and the GC-detectable compounds.

### 3.4.2 Lignin-Derived Substances

GC/MS analysis showed that lignin-derived monomers in a low amount were present in the extracts, indicating that these compounds were at least partly soluble in *n*-heptane. The main part of the lignin-derived components present in the FPBOs are nonpolar and are known to contain monomers and oligomeric aromatic units.^31^ However, only lignin-derived monomers and some dimers can be analyzed by GC. Hence, UV spectroscopy at 280 nm was used to estimate the content of lignin-derived substances in the *n*-heptane extract (a sample from the 4 h shaker with a stir bar). Based on the analysis, 70 ± 7 wt % of the *n*-heptane extract was composed of lignin-derived substances. Lipophilic extractives are mostly composed of nonaromatic compounds; an exception is dehydroabietic acid found in the top phase (0.05 wt %). Hence, it was concluded that a major part of the *n*-heptane-soluble compounds was composed of lignin-derived substances.

## 4 Conclusions

The extractive-derived substances in FPBOs, especially from forest residues, form a separate layer with char particles on the surface of these oils. In this study, a proper analysis method for these extractives was developed. The most important findings were as follows:

*n*-Heptane and *n*-hexane were more selective solvents for fatty acids, resin acids, and terpenoids than more polar solvents, such as MTBE, EtOAc, *t*-BuOH, and DCM, which dissolved more lignin monomers and anhydrosugars together with non-GC-detectable substances. Because of the similar solubility properties of *n*-hexane and *n*-heptane and the less toxic nature of *n*-heptane, *n*-heptane was recommended instead of *n*-hexane. Extraction in a shaker for 2 h with a stir bar using a sample-to-solvent ratio of 1:30 was found to be a straightforward and fast method for the reproducible separation of extractives and in high yield from the other bio-oil components.

The GC and UV spectroscopic analyses showed that the *n*-heptane extract was mainly composed of extractives (27 wt %) and lignin-derived compounds (70 wt %). Hence, the extraction of the FPBO top phase with *n*-heptane followed by this analysis practice was the useful way to estimate the content and composition of lipophilic extractives.

## Abbreviations

*t*-BuOH: *tert*-butanol
BSTF: *N*,*O*-bis(trimethylsilyl)trifluoroacetamide
DCM: dichloromethane
EtOAc: ethyl acetate
FID: flame ionization detector
FPBO: fast pyrolysis bio-oil
GC: gas chromatography
MS: mass spectrometry
MSD: mass selective detector
MTBE: *tert*-methyl butyl ether
TMAAc: tetramethyl ammonium acetate
TMAH: tetramethyl ammonium hydroxide
TMCS: trimethylchlorosilane
UV: ultraviolet
